# Establishment of Human Papillomavirus Infection Requires Cell Cycle Progression

**DOI:** 10.1371/journal.ppat.1000318

**Published:** 2009-02-27

**Authors:** Dohun Pyeon, Shane M. Pearce, Simon M. Lank, Paul Ahlquist, Paul F. Lambert

**Affiliations:** 1 McArdle Laboratory for Cancer Research, University of Wisconsin-Madison, Madison, Wisconsin, United States of America; 2 Institute for Molecular Virology, University of Wisconsin-Madison, Madison, Wisconsin, United States of America; 3 Howard Hughes Medical Institute, University of Wisconsin-Madison, Madison, Wisconsin, United States of America; Brigham and Women's Hospital and Department of Medicine, United States of America

## Abstract

Human papillomaviruses (HPVs) are DNA viruses associated with major human cancers. As such there is a strong interest in developing new means, such as vaccines and microbicides, to prevent HPV infections. Developing the latter requires a better understanding of the infectious life cycle of HPVs. The HPV infectious life cycle is closely linked to the differentiation state of the stratified epithelium it infects, with progeny virus only made in the terminally differentiating suprabasal compartment. It has long been recognized that HPV must first establish its infection within the basal layer of stratified epithelium, but why this is the case has not been understood. In part this restriction might reflect specificity of expression of entry receptors. However, this hypothesis could not fully explain the differentiation restriction of HPV infection, since many cell types can be infected with HPVs in monolayer cell culture. Here, we used chemical biology approaches to reveal that cell cycle progression through mitosis is critical for HPV infection. Using infectious HPV16 particles containing the intact viral genome, G1-synchronized human keratinocytes as hosts, and early viral gene expression as a readout for infection, we learned that the recipient cell must enter M phase (mitosis) for HPV infection to take place. Late M phase inhibitors had no effect on infection, whereas G1, S, G2, and early M phase cell cycle inhibitors efficiently prevented infection. We conclude that host cells need to pass through early prophase for successful onset of transcription of the HPV encapsidated genes. These findings provide one reason why HPVs initially establish infections in the basal compartment of stratified epithelia. Only this compartment of the epithelium contains cells progressing through the cell cycle, and therefore it is only in these cells that HPVs can establish their infection. By defining a major condition for cell susceptibility to HPV infection, these results also have potentially important implications for HPV control.

## Author Summary

Human papillomaviruses (HPV), which comprise more than 100 genotypes, are the most prevalent sexually transmitted infection and are associated with multiple human cancers including all cervical cancers, many other anogenital cancers, and 25% of head and neck cancers. The HPV life cycle is closely linked to epithelial differentiation of skin keratinocytes, with initial infection occurring only in the undifferentiated proliferating basal compartment of the epithelium and progeny virus production only in the terminally differentiated suprabasal compartment. So far, little is known about how host cells restrict the HPV life cycle to specific stages of skin cell development. Here, by identifying small molecule inhibitors of HPV infection, we discovered that cell cycle progression through mitosis is critical for the establishment of HPV infection. In addition, our further chemical genetic dissection of this process showed that early steps of mitosis are required for HPV infection and early gene expression. Our findings provide one reason why HPV only infects undifferentiated proliferating cells and provide new leads for the development of preventive and therapeutic strategies against HPV infection.

## Introduction

Papillomaviruses (HPV) are small, non-enveloped, double stranded DNA viruses that infect the cutaneous and/or mucosal epithelium in many vertebrates [1]. Over 100 human papillomavirus (HPV) genotypes are categorized as high risk (HPV16 and HPV18) or low risk (HPV6 and HPV11) depending on their oncogenicity. High risk genotypes are causally associated with anogenital cancers including nearly 100% of cervical carcinomas, the second leading cause of death from cancer in women worldwide [1]–[3]. HPV is also associated with ∼25% of head and neck cancers, the sixth most common cancer in the United States [4],[5].

The HPV life cycle is tightly linked to the differentiation of its host epithelial cells [6],[7]. This complex life cycle includes a) infection and establishment of the viral DNA as a multicopy nuclear plasmid, which only occurs in dividing basal cells of stratified epithelium; b) maintenance of the viral DNA at low copy number in dividing basal epithelial cells; and c) high copy number DNA amplification and encapsidation in non-dividing, terminally differentiated epithelial cells to yield progeny virions. HPV early genes are expressed throughout this life cycle, while late capsid genes L1 and L2 are only expressed in terminally differentiated epithelial cells. The mechanisms controlling this life cycle, particularly the restriction of HPV infection and DNA establishment to dividing basal cells, are not well understood.

The tight link between keratinocyte differentiation and the HPV life cycle has made large scale production of mature infectious HPV particles difficult, greatly restricting studies of the mechanisms of natural HPV infection [8],[9]. Recently developed transfection methods that generate large yields of virus particles and efficient encapsidation of target plasmids as large as the full length ∼8 kb HPV genome have overcome this limitation 10,11. This technique provides a genetically modifiable, high yield source of infectious HPV and HPV pseudoviruses expressing reporter genes for studies of different early steps of HPV infection including virion binding, endocytosis, uncoating of the virion capsid, release from the endosome, trafficking to the nucleus, delivery of viral DNA to the nucleus, and transcription of encapsidated genes.

To define the pathways and mechanisms involved in these early steps of HPV infection, we tested approximately 5,000 bioactive compounds with known mechanisms of action for effects on the entry of HPV capsids containing reporter genes or the full HPV genome, and identified a subset of cell cycle inhibitors that completely blocked wild type HPV infection. Our further studies showed that cell cycle progression through early stages of mitosis is critical for successful HPV infection. These findings reveal new insights into the mechanism by which HPV infects cells and provide one reason why HPV infects only undifferentiated, proliferating cells. These results also provide new leads for developing preventive and therapeutic strategies against HPV infection.

## Results

### Cell cycle inhibitors block HPV infection

To identify mechanistic pathways and informative modulators of HPV infection, we tested nearly 5,000 compounds in known bioactive molecule libraries from Prestwick (Prestwick Chemicals) and LOPAC (Library of Pharmacologically Active Compounds, Sigma), using HPV pseudovirions containing an SV40 promoter-driven secreted alkaline phosphatase (SEAP) reporter gene (*hpv*SEAP). High activity effectors identified in this assay were retested in a second infectivity assay using HPV pseudovirions containing renilla luciferase driven by the natural HPV16 promoter (*hpv*16wpA-RL) to exclude false positive compounds that may selectively affect the SV40 promoter or alkaline phosphatase (data not shown). This screening assay was performed in human kidney 293T cells owing to the high signal to noise ration, and resulting high z scores obtained in this cell line. Secondary screens performed in HaCaT cells, an immortalized line of human keratinocytes, which are natural host cells for HPV infection, confirmed the validity of the hits, as indicated below.

**Figure 1:**
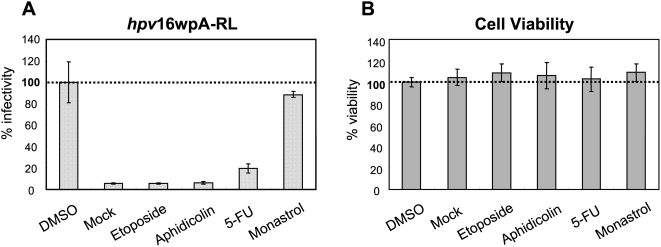
Selected cell cycle inhibitors abrogate HPV infection. The reporter RL activity (A) and cell viability (B) were measured by Renilla Luciferase Assay System (Promega) and CellTiter-Glo Luminescent cell viability assay (Promega), respectively. Human keratinocyte HaCaT cells were inoculated with HPV16 LCR-driven RL containing pseudovirion *hpv*16wpA-RL after 4 hr pre-treatment with 3 µM of each compound, and reporter expression/cell viability scored 48 hours later. The expression value is shown as % infectivity or % viability to untreated cells with *hpv*16wpA-RL.

Among the confirmed hits identified in the primary screen were cell cycle inhibitors etoposide [12] and aphidicolin [13], which showed the most significant and consistent inhibition of HPV infection in 293 cells both using HPV16 pseudoviruses expressing SEAP or renilla luciferase reporter genes (data not shown), or using intact infectious HPV16 virus in which we scored for early gene expression (see Figure S1). The levels of inhibition achieved with these drugs was greater than that achieved with neutralizing antibody to HPV16 (Figure S1A), and were specific to virally expressed gene as the drugs had no effect on cellular β-actin expression (Figure S1B). In the more physiologically relevant HaCaT cells, these two drugs, along with another cell cycle inhibitor 5-fluorouracil (5-FU) [14], all were very robust in inhibiting infectivity (Figure 1A) at concentrations that did not have any effect on cell viability (Figure 1B). These results demonstrate that the original screen, which was done in 293T cells, was successful in identifying drugs that can inhibit HPV infection in a more relevant cell type. More importantly our results suggested that the cell cycle is critical for HPV infection.

**Figure 2:**
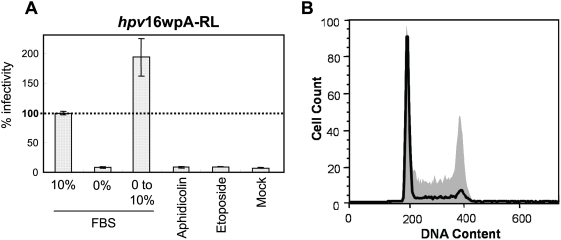
Cell cycle arrest by serum starvation abrogates HPV infection. (A) After 24 hr incubation in serum-free DMEM, HaCaT cells were inoculated with HPV pseudovirion *hpv*16wpA-RL, and RL activity was measured after 2 d. For serum conversion, serum-free DMEM was replaced with 10% FBS/DMEM before virus inoculation. The expression value is shown as % infectivity to untreated cells with *hpv*16wpA-RL inoculation. *Columns*, mean; *bars*, SD. (B) Flow cytometry was performed to confirm cell cycle arrest by serum starvation. The solid line indicates the HaCaT cells incubated with serum-free DMEM for 24 h, and the gray shaded area indicates control HaCaT cells in DMEM with 10% FBS.

To ensure that the negative effects of these drugs on early steps in HPV infection were due to their inhibition of the cell cycle, and not other activities such as the induction of DNA damage responses, we arrested the cell cycle using a non-chemical method, serum starvation. HaCaT cells incubated in serum free medium showed complete cell cycle arrest at G1 phase and dramatic inhibition of HPV infection (Figure 2A and 2B). However, when the cell cycle was released by serum addition at the time of HPV inoculation, HPV infection was not inhibited, but enhanced about two-fold. These results confirm that cell cycle progression is necessary for HPV early infection and viral gene expression.

### Cell cycle progression through mitosis is critical for HPV infection

**Figure 3:**
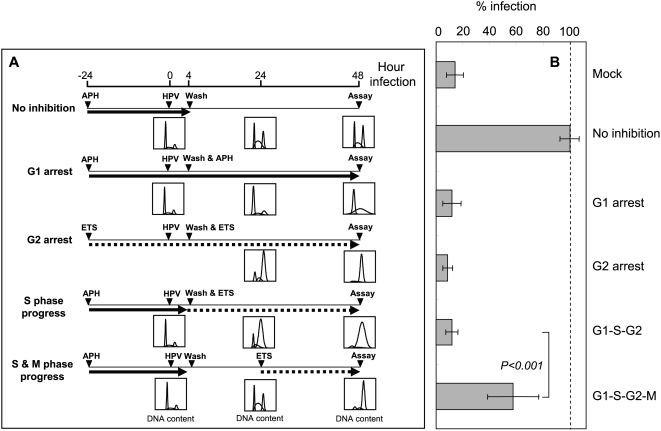
Cell cycle progression through M phase is critical for HPV infection. (A) HaCaT cells were synchronized with aphidicolin (sets 1, 2, 4, and 5) and etoposide (set 3) 24 hrs before *hpv*16wpA-RL inoculation. Cell cycle progression was released in set 1 and kept blocked in set 2 and 3 using aphidicolin and etoposide, respectively. In set 4, cell cycle progressed through S phase but arrested at G2 phase by switching the compounds in medium from aphidicolin to etoposide. In set 5, cell cycle was released for 20 hrs to progress through M phase and arrest at G2 phase by etoposide addition. Experimental protocols are shown as diagrams. Solid arrow and dotted arrow indicate aphidicolin and etoposide in cell culture medium, respectively. Histograms show the results of flow cytometry analysis following propidium iodide staining at 0, 24, and 48 hr time points using a cell cycle analysis program of FlowJo. (B) The reporter RL activity was measured by Renilla Luciferase Assay System (Promega).

**Figure 4:**
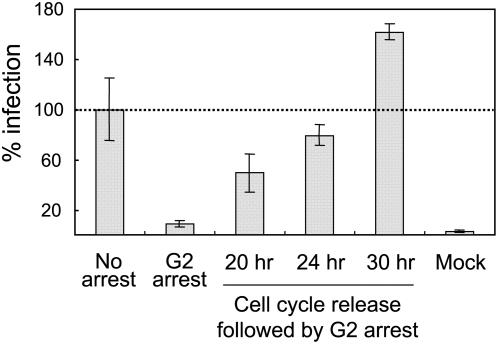
Cell cycle progression through M phase is critical for HPV infection. Cell cycle release after 24 hr G1 arrest was extended to 24 and 30 hrs before G2 arrest by etoposide. Expression of HPV16 early RNA transcripts was measured using E7 specific primers. *Columns*, mean; *bars*, SD. We could not detect any signal above the background in reverse transcriptase–negative controls, indicating that only mRNA expressed from infected virus could be detected (data not shown).

To define which cell cycle step(s) is critical for early HPV infection, we synchronized HaCaT cells with aphidicolin or etoposide and then tightly controlled subsequent cell cycle progression. In the first set of experiments (indicated as “No inhibition”; Figure 3A and 3B), HaCaT cells were synchronized in G1 phase by a 24 hr aphidicolin treatment, inoculated with *hpv*16wpA-RL virions, and then 4 hr later, released by removing aphidicolin from the culture medium. After 48 hr incubation, the infected HaCaT cells expressed high levels of RL. The reporter gene expression levels under these “no inhibition” positive control conditions were set to 100% infection and used to normalize the early gene expression levels from the other experimental conditions (Figure 3B). In the second (G1 arrest; Figure 3A and 3B) and third (G2 arrest; Figure 3A and 3B) conditions used, cells were treated with either aphidicolin or etoposide for 24 hr, after which *hpv*16wpA-RL virions were added. The cell cycle was blocked at G1/S and G2 by aphidicolin and etoposide, respectively, and HPV infection was significantly and consistently inhibited under both conditions (Figure 3A and 3B). These conditions were the same as used in Figure 1, and the flow cytometric analyses shown in Figure 3 confirmed effective cell cycle block by these aphidicolin and etoposide at the concentrations used in Figure 1. In the fourth condition (S phase progress; Figure 3A and 3B), we synchronized the cells in G1/S phase by 24 hr aphidicolin treatment, then added *hpv*16wpA-RL virions and, 4 hr later, replaced aphidicolin with etoposide. When aphidicolin was replaced by etoposide, cell cycle progressed from G1 arrest through S phase to G2 arrest, allowing us to test whether S phase progression supports HPV early infection. However, this experiment showed no difference in reporter gene expression from G1/S or G2 arrest by aphidicolin and etoposide (Figure 3A and 3B). In the fifth condition (S & M phase progress; Figure 3A and 3B), after 24 hr synchronization and subsequent *hpv*16wpA-RL virion addition, the cell cycle was released for 20 hrs, followed by cell cycle arrest in G2 phase with etoposide, allowing the cells to progress through one complete round of the cell cycle, including M phase. Interestingly, one round of M phase progression was sufficient for HPV infection and gene expression (Figure 3A and 3B). Our further testing with wild type HPV16 virions confirmed that one round of the cell cycle through M phase is sufficient for HPV early gene expression (Figure S2). These results imply that cell cycle progression through mitosis is critical for early steps of HPV infection. Consistent with this premise, HPV gene expression levels were further enhanced when the cell cycle was released for progressively longer periods before G2 arrest, eventually reaching levels well above the control “no arrest” conditions (Figure 4). This suggests that G2 arrest following a complete cell cycle might provide a better host cell environment for viral gene expression once the virus enters nucleus.

### Early prophase, but not late prophase or metaphase, is critical for HPV early infection

**Figure 5:**
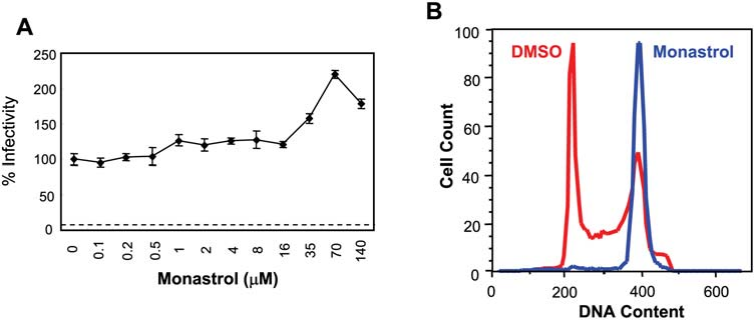
Late M phase arrest does not affect HPV infection. (A) HaCaT cells were treated with different concentrations of monastrol, and *hpv*16wpA-RL infectivity was assessed as above. Dotted line is for mock-infected cells. (B) Cell cycle arrest at M phase by monastrol (100 µM) was confirmed using flow cytometry. Red and blue histograms indicate the cell cycle status of DMSO and monastrol-treated cells, respectively.

**Figure 6:**
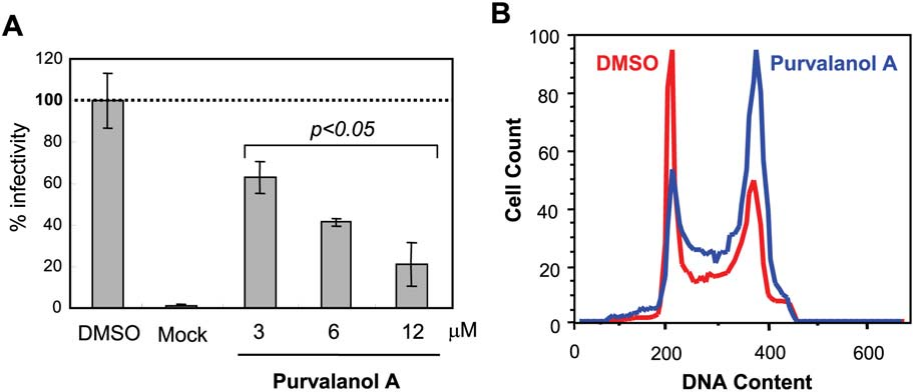
Early M phase arrest by CDK1 inhibition interferes with HPV infection. (A) 293T cells were treated with a CDK1 inhibitor, purvalanol A (3, 6, and 12 µM), and inoculated with *hpv*SEAP. SEAP activity was assayed after 48 hrs incubation as described in Materials and Methods. p<0.05, significantly different from DMSO control. *Columns*, mean; *bars*, SD. (B) Cell cycle arrest at M phase by purvalanol A (12 µM) is shown. Red and blue histograms indicate the cell cycle status of DMSO and purvalanol A-treated cells, respectively.

To define further the stages of mitosis and associated cellular functions that are or are not required to support HPV early infection, we tested monastrol [15], which inhibits mitotic spindle formation during late prophase and metaphase in early to mid mitosis. Interestingly, monastrol, which induced cell cycle arrest in M as expected (Figure 5B) showed no inhibitory effect on HPV infection in HaCaT cells (Figure 5A). Indeed at high concentrations of monastrol, there was a slight but reproducible increase in HPV infectivity. Thus HPV infection can efficiently arise in cells inhibited in late prophase/metaphase. These results, taken together with those in Figure 3 narrowed down the critical stage of the cell cycle for HPV infection to likely be early prophase. Cellular events in early prophase include nuclear envelope (NE) breakdown and changes in chromatin structure. Phosphorylation of NE components by CDK1 is critical for these events in early prophase [16]–[18]. To examine whether entry into early prophase is critical for HPV infection, we tested *hpvSEAP* infection in 293T cells treated with the CDK1 inhibitor, purvalanol A [19]. Purvalanol A inhibited HPV infection dose-specifically, with 12 µM both significantly arresting the cell cycle at G2/M phase and inhibiting HPV infection ∼5-fold (Figure 6A and 6B). These results imply that cellular event(s) during early prophase are critical for HPV infection.

### Influenza virus genome delivery via nuclear pores is unaffected by cell cycle inhibitors that block HPV infection

**Figure 7:**
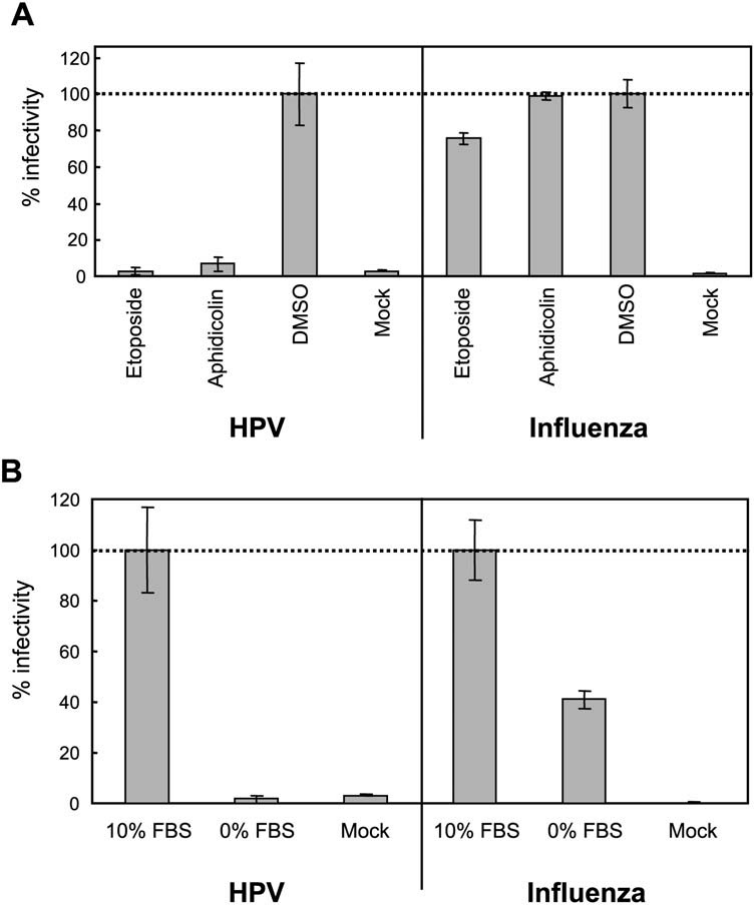
Cell cycle arrest does not inhibit influenza virus. (A) After 24 hr synchronization with aphidicolin and 4 hr treatment with etoposide or aphidicolin (3 µM each), 293T cells were inoculated in parallel with *hpv*16wpA-RL or an influenza virus vector in which the hemagglutinin and neuraminidase open reading frames in viral RNA were replaced with those of vesicular stomatitis virus glycoprotein and RL, respectively [50]. (B) After 24 hr synchronization in serum-free DMEM, HaCaT cells were inoculated with *hpv*16wpA-RL and the RL-expressing influenza virus as (A) in the presence or absence of FBS. RL activity was measured after 48 hrs, as described in Materials and Methods, normalized to RL activity in equivalently inoculated cells maintained in 10% FBS, and expressed in the histograms as % infectivity. *Columns*, mean; *bars*, SD.

HPV minor capsid protein L2 has nuclear localization signals and, consequently, it has been hypothesized that L2 direct nuclear pore-mediated import of HPV DNA during infection [20]. One virus that is well established to import its genome into intact nuclei via nuclear pores is influenza virus [21],[22]. To test whether HPV and influenza virus share similar nuclear entry mechanisms, we treated 293T cells in parallel with either a recombinant influenza virus expressing renilla luciferase or renilla luciferase-expressing HPV pseudovirions, in the presence and absence of etoposide and aphidicolin. While etoposide and aphidicolin efficiently blocked HPV infection, as before, they did not significantly affect influenza virus infection (Figure 7A), showing that their effects on HPV could not be explained by effects on import via nuclear pores. We also observed in HaCaT cells the same difference in cell cycle dependency when those cells were arrested by serum starvation and inoculated with the same renilla luciferase-expressing HPV pseudovirions or recombinant influenza virus. Under these conditions (Figure 7B), HPV infection was inhibited over 50 fold while influenza virus infection was only reduced approximately 2-fold, likely due to the general suppression of cell metabolism upon serum starvation.

## Discussion

HPVs are pathogens of tremendous clinical importance due to their strong associations with multiple common human malignancies and because they are the most common sexually transmitted pathogens. Some oncogenic mechanisms of HPV are well known, especially those of viral oncoproteins E6 and E7 [23]. However, many other basic mechanisms of the HPV life cycle are largely unknown including how HPV selectively infects only undifferentiated basal cells. In this report, we define one of the bases for this restriction by showing that cell cycle progression into mitosis and, in particular, events in early prophase are critical for establishing HPV infection.

Viruses employ varied strategies to deliver their genetic material to the nucleus for replication and viral gene expression [24]. While adenoviruses dissociate their capsids in the cytoplasm and import naked genomic DNA into the nucleus, herpesvirus virions release their genome into the nucleus without any extensive capsid disassembly, and polyomaviruses and parvoviruses transport the entire capsid into the nucleus. The nuclear import strategies of papillomaviruses have been largely unexplored, mainly because the means for producing high yields of infectious virus have only recently been attained [10],[11]. In the present study, using infectious wild type HPV16 virions, we found that events in the early prophase segment of mitosis are critical for establishing HPV infection, as assayed by introduction and expression of HPV-encapsidated DNA in the nucleus. As discussed below, these events could include nuclear envelope breakdown, cytoskeleton restructuring, and subnuclear structure changes as well as the specific expression of one or more genes or gene combinations in early mitosis.

One critical event in early mitosis is nuclear envelope (NE) breakdown, which is triggered by a signal cascade that involves polo I like kinase 1 (plk1) and cyclin B1-associated CDK1 [16],[17],[25]. Using a chemical inhibitor of CDK1, purvalanol A [19], we found that CDK1 activity is required for efficient infection by HPVs (Figure 6A). Therefore, our data are consistent with the possibility that NE breakdown is necessary for the HPV encapsidated DNAs to enter the nucleus. This is similar to the strategy thought to be employed by simple retroviruses to allow nuclear entry and integration of their proviral genomes [26],[27]. The process that initiates nuclear envelope breakdown is not fully understood. While the phosphorylation of NE components by CDK1 correlates with NE breakdown, and inhibition of cdk1 prevents NE breakdown [16],[17], some have argued that NE breakdown is initiated by mechanical tension induced by spindle microtubules, leading to holes in the NE [28],[29]. Consistent with this hypothesis, the microtubule minus end motor, dynein, translocates to the outer face of the NE just before NE breakdown. This model for NE breakdown by mechanical tension has an interesting connection to HPV infection. L2 binds to dynein and this is thought to allow L2∶HPV DNA complexes to be translocated along microtubules towards the nucleus [30]. Thus, dynein-mediated trafficking of HPV-encapsidated DNA cargo may not just contribute to movement of the encapsidated DNA to the intracytoplasmic destination of dynein, the microtubule organizing center, but might also contribute to nuclear localization if the above described model for NE breakdown is correct.

Another notable cellular event in early mitosis is the reorganization of cytoplasmic microtubules to support mitotic spindle formation, chromosome segregation, and cell division [31],[32]. Like many other viruses, HPV utilizes microtubules to traffic from the cell surface to the nucleus [30], although many details of this process remain to be understood. Our results are consistent with the possibility that effective HPV entry and/or HPV DNA delivery require cell cycle-associated reorganization of microtubules, such as the interaction of microtubules with host chromosomes in early mitosis.

In addition, restructuring of PML oncogenic bodies (PODs) and chromatin in early mitosis could be necessary for establishing HPV infection in the nucleus. PODs, also known as ND10, are subnuclear bodies implicated in multiple cellular functions including transcription, DNA repair, viral defense, stress, cell cycle regulation, proteolysis and apoptosis [33],[34]. PODs are also the final destination of HPV DNA during the initial steps in infection and sites at or near which HPV DNA replication and transcription occur [35],[36]. PODs are dynamically restructured during S and M phases, responding to changes in chromatin organization [37]. Thus, structural changes of PODs in early mitosis might be critical for HPV DNA localization to its POD destinations during the establishment of HPV infection. Other host mechanisms associated with chromatin structure in early mitosis might also contribute to structural changes of the incoming HPV DNA and thereby facilitate viral gene expression and replication inside the nucleus. Little is currently known about HPV chromatin structure and its contribution to viral gene expression.

Many genes such as polo-like kinases and aurora kinases are exclusively expressed in early mitosis to certify completion of DNA replication, overcome mitotic checkpoint, and initiate mitosis [38]. Beyond the potential mechanisms of cell cycle dependence suggested above, host genes or gene combinations specifically expressed only in early mitosis might have essential roles for HPV entry steps and/or HPV early gene expression.

HPV is thought to be able to infect the proliferating basal layer of stratified epithelium only when wounds allow HPV to penetrate the physical barrier of upper skin layers. Wounding also can provide HPV particles access to laminin 5 or heparin sulfate moieties, both components of the extra cellular matrix (ECM) component of the epithelial basement membrane for which HPV particles have high affinity, thereby localizing the virus particles to basal surface of epithelial cells it needs to infect [39],[40]. Laminin 5 is also the ECM partner of integrin α6/β4, which has been reported by some investigators to be an entry receptor for HPVs [39]–[41]. Our results suggest that wound healing might increase the efficiency with which the HPV DNA becomes established as a nuclear plasmid in basal cells, because the basal cells are then in a hyperproliferative state [42]. HPV may also provide a mitogenic signal upon binding to cell surface receptors [43],[44], though if so, that signal is insufficient to overcome serum starvation-induced cell cycle arrest in HaCaT cells (Figure 2A and 2B). Regardless of why the cell is cycling, our data indicate that its movement through mitosis is one critical step in establishing HPV infection. To further confirm physiologically relevance regarding the cell cycle dependence of HPV infection, it would be necessary to examine these results in primary keratinocytes and *in vivo* models such as mouse and non-human primate. In addition, real-time particle imaging studies could be employed to identify what specific steps in infection are blocked upon cell cycle arrest; however, the ability to interpret accurately such imaging studies rests on the ability to distinguish particles leading to infectious events from the vast majority of particles that give rise to abortive or non-infections events. To date no imaging method exists able to make such a distinction at the individual particle level.

The recent development of HPV vaccines offers an extremely important avenue for control of HPV infections and their associated cancers [45]. Nevertheless, due to limitations of recently approved HPV vaccines, including incomplete coverage of high-risk HPV genotypes and high cost, there is an urgent need to identify other approaches to prevent HPV infections. One potentially valuable approach to prevent STDs, including genital HPV infections, could be ready access to effective microbicides [46]. From this perspective, our findings provide a new target mechanism for preventing initial HPV infection and blocking further spread. Recently, Schiller's group has developed an *in vivo* mouse model of HPV infection in mouse reproductive organs [47]. Using this method, our small molecule HPV inhibitors are now undergoing tests to assess their efficacy and therapeutic potential. In this context, our high throughput chemical biology screening approach that led to the current study on the role of cell cycle in HPV infection also has successfully identified many other compounds that inhibit HPV infection, and their initial evaluation points to promising lead compounds for further development as microbicides. It remains to be seen whether establishment of a robust high throughout screen using human keratinocytes instead of a surrogate cell type, here 293T cells, could identify additional small molecules and possibly other cellular processes critical for HPV infection in its natural host.

## Materials and Methods

### Plasmids

pEF399, containing the complete W12E HPV16 genome [48], and all other plasmids used for virus packaging were previously described [11]. pSEAP (pSEAP-control) for expression of secreted alkaline phosphatase (SEAP) was purchased from Clontech. pHPV16wpA-RL was cloned with HPV16 long control region (LCR, nucleotide position 7155 - 861), into pRL-null (Promega) to prepare a Renilla luciferase reporter system driven by native HPV16 promoter.

### Cell lines

Human embryonic kidney cell line 293T from ATCC, and its enhanced SV40 T antigen-expressing daughter cell line 293TT [10] from John Schiller, were maintained in Dulbecco's modified eagle's medium (DMEM) supplemented with 10% fetal bovine serum (FBS) (Invitrogen). Immortalized human keratinocyte cell line HaCaT [49] was maintained in F-media (Invitrogen, 3 parts F-12 and 1 part DMEM), supplemented with 10% FBS.

### Production of virus particles

HPV virions and pseudovirions were prepared as described previously [11]. Briefly, we cotransfected 293TT (provided by John Schiller) cells with HPV16 capsid protein expression plasmid as well as one of the target DNAs for encapsidation. After 48 h at 37°C, cells were harvested and virions were purified using Optiprep gradient centrifugation. Influenza virus containing RL [50] was provided by Yoshihiro Kawaoka.

### Small molecule compounds and libraries

Known Bioactive Library (KBA01) consists of 3 commercially available collections totaling 4,160 compounds. KBA01 consists of 880 high purity compounds of known safety and bioavailability in humans of which over 85% are marketed drugs from Prestwick Chemical. The Prestwick compounds cover several therapeutic areas including neuropsychiatry, cardiology, immunology, inflammation, analgesia, etc. Also included in the KBA01 library is 2000 diverse FDA approved drugs and natural products from Spectrum Chemical Collection From Microscource Discovery Systems, Inc. and 1280 compounds from the LOPAC of Sigma representing marketed drugs, failed development candidates and “gold standards” that have well-characterized activities. These compounds are the results of lead optimization efforts and have been rationally designed by structure-activity relationship studies. The NCI03 library consists of 235 natural products that were selected from the NCI open repository on the basis of structural diversity and availability of compound. Etoposide, Aphidicolin, Monastrol, 5-Fluorouracil, CGP-74514A were purchased from Sigma.

### High throughput screening

293TT cells in 384-well plates were treated with 3.3 µM of library compounds for 4 hrs and inoculated with *hpv*SEAP at 50 vge (viral genome equivalent)/cell for 48 hrs. Culture supernatant 2.5 µl was used for virus infectivity assay using a Phospha-Light a chemiluminescent alkaline phosphatase assay (Applied Biosystems) and cell lysate was used for cell viability assay using CellTiter-Glo Luminescent cell viability assay (Promega). Biomek FX (Beckman Coulter) was used for automated liquid handling and a Victor 3-V plate reader (Perkin Elmer) was used for measuring luminescence. Screening was performed in the Small Molecule Screening Facility at the University of Wisconsin Comprehensive Cancer Center.

### Infectivity assay

The activity of pSEAP-control-encapsidated pseudovirions (*hpv*SEAP) was measured using a Phospha-Light a chemiluminescent alkaline phosphatase assay (Applied Biosystems) and the activity of pHPV16wpA-RL-encapsidating pseudovirions (*hpv*16wpA-RL) was assayed using Renilla Luciferase Assay System (Promega). Infectivity of wild type HPV16 virions was examined by quantitative reverse transcriptase PCR (qRT-PCR) by amplifying viral mRNA signals from HPV16-treated 293TT cells. 293TT cells were inoculated with 50–100 vge/cell of packaged virus and incubated for 48 h at 37°C. Total RNA was isolated using the RNeasy total RNA purification kit (Qiagen), treated with RQ DNaseI (Promega) to remove possible DNA contaminants, purified again on RNeasy columns to remove DNaseI, and quantified by spectrophotometer. cDNA was synthesized from 20 µg of total RNA with oligo (dT) using a SuperScript cDNA synthesis kit (Invitrogen), and qPCR was performed with QuantiTect SYBR Green PCR Kit (Qiagen). Oligonucleotide primers (Figure 3) were designed using the Primer3 primer design program [51], synthesized by MWG and used at 0.5 µM for PCR amplification for 40 cycles of 30 s denaturation at 94°C, 30 s annealing at 55°C, and 30 s polymerization at 72°C. Obtained values were normalized with the levels of β-actin.

### Flow cytometry

The cell cycle status of treated and untreated cells was analyzed using a propidium iodide (PI) incorporation method. Briefly, HaCaT cells were harvest, homogenized, fixed with cold 70% ethanol for 30 min at −20°C, and incubated with PI staining solution (1 mg/ml RNase A, 33 µg/ml PI, 0.2% NP-40 in PBS) for 30 min at room temperature. Stained cells were filtered through 35 µm-pore nylon mesh cell filtering caps (Falcon) and analyzed by flow cytometry using Becton Dickinson FACSCalibur (488 nm laser excitation).

## Supporting Information

Figure S1 Selected cell cycle inhibitors abrogate HPV infection. HPV16 early gene (A) and β-actin expression (B) was measured by quantitative RT-PCR. 293T cells were inoculated with wild type HPV16 after 4 hr pre-treatment with 3 µM of each compound or with 1∶100 dilution of neutralizing antibody (H16.7E). Total RNA was extracted after 48 hrs, and expression levels of HPV16 RNA transcripts were measured by qRT-PCR using E7, E2, and E5 sequence specific primers (Figure S3). The expression value is shown as % infectivity or % expression to untreated cells infected with HPV16. We could not detect any signal above the background in reverse transcriptase-negative controls, indicating that only mRNA expressed from infected virus could be detected (data not shown). Due to different characteristics of each primer set, we observed slight differences in the quantity of mRNAs detected in the same sample with different primer pairs. Those differences, while not statistically significant, could reflect differences in the efficiency of each primer pair amplifying different subsets of viral early mRNAs. Veratrine, an alkaloid drug, was one of many small molecule compounds in the initially screened libraries that did not affect HPV infection and was chosen as an irrelevant negative control compound for the secondary screens. The neutralizing antibody (H16.7E, provided by Neil Christensen at the Pennsylvania State University, Hershey, Pennsylvania) was used as a positive control for HPV infection inhibition.
(0.12 MB PDF)

Figure S2 Cell cycle progression through M phase is critical for HPV infection. 293T cells were synchronized with aphidicolin (sets 1, 2, 4, and 5) and etoposide (set 3) 4 hrs before wild type HPV16 inoculation. Cell cycle progression was released in set 1 and kept blocked in sets 2 and 3 using aphidicolin and etoposide, respectively. In set 4, cell cycle progressed through S phase but arrested at G2 phase by switching the compounds in medium from aphidicolin to etoposide. In set 5, cell cycle was released for 20 hrs to progress through M phase and arrest at G2 phase by etoposide addition. Wild type HPV16 gene expression was measured from total RNA extracts as indicated in Figure S1 and in Materials and Methods.
(0.06 MB PDF)

Figure S3 Quantitative PCR primers of HPV16 early genes. (A) Oligonucleotide primers were designed using the Primer3 primer design program. (B) The HPV genomic position of each primer is indicated by an arrow.
(0.01 MB PDF)
