# Supplementary material for: Establishment of Human Papillomavirus Infection Requires Cell Cycle Progression
Source: PLoS Pathog. 2009 Feb 27;5(2):e1000318. doi: 10.1371/journal.ppat.1000318 (PMC2642596; doi:10.1371/journal.ppat.1000318)
Supplement: Figure S1 — Selected cell cycle inhibitors abrogate HPV infection. HPV16 early gene (A) and β-actin expression (B) was measured by quantitative RT-PCR. 293T cells were inoculated with wild type HPV16 after 4 hr pre-treatment with 3 µM of each compound or with 1∶100 dilution of neutralizing antibody (H16.7E). Total RNA was extracted after 48 hrs, and expression levels of HPV16 RNA transcripts were measured by qRT-PCR using E7, E2, and E5 sequence specific primers (Figure S3). The expression value is shown as % infectivity or % expression to untreated cells infected with HPV16. We could not detect any signal above the background in reverse transcriptase-negative controls, indicating that only mRNA expressed from infected virus could be detected (data not shown). Due to different characteristics of each primer set, we observed slight differences in the quantity of mRNAs detected in the same sample with different primer pairs. Those differences, while not statistically significant, could reflect differences in the efficiency of each primer pair amplifying different subsets of viral early mRNAs. Veratrine, an alkaloid drug, was one of many small molecule compounds in the initially screened libraries that did not affect HPV infection and was chosen as an irrelevant negative control compound for the secondary screens. The neutralizing antibody (H16.7E, provided by Neil Christensen at the Pennsylvania State University, Hershey, Pennsylvania) was used as a positive control for HPV infection inhibition. (0.12 MB PDF) [file ppat.1000318.s001.pdf]

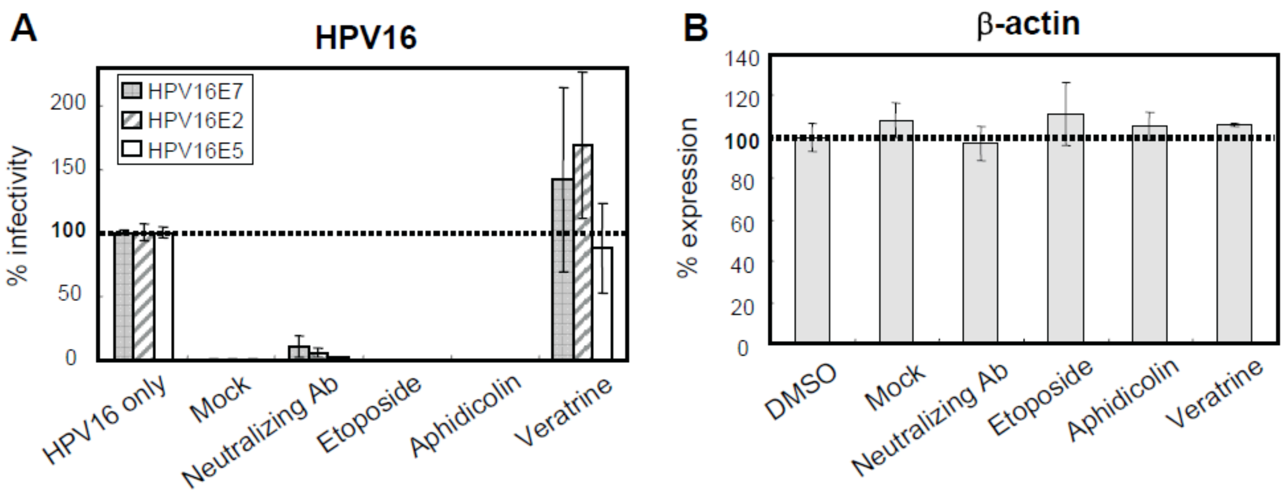

Supplementary Figure 1. Selected cell cycle inhibitors abrogate HPV infection. HPV16 early gene (A) and β-actin expression (B) was measured by quantitative RT-PCR. 293T cells were inoculated with wild type HPV16 after 4 hr pre-treatment with 3 μM of each compound or with 1:100 dilution of neutralizing antibody (H16.7E). Total RNA was extracted after 48 hrs, and expression levels of HPV16 RNA transcripts were measured by qRT-PCR using E7, E2, and E5 sequence specific primers (Supplementary Figure 3). The expression value is shown as % infectivity or % expression to untreated cells infected with HPV16. We could not detect any signal above the background in reverse transcriptase-negative controls indicating that only mRNA expressed from infected virus could be detected (data not shown). Due to different characteristics of each primer set, we observed slight differences in the quantity of mRNAs detected in the same sample with different primer pairs. Those differences, while not statistically significant, could reflect differences in the efficiency of each primer pair amplifying different subsets of viral early mRNAs. Veratrine, an alkaloid drug, was one of many small molecule compounds in the initially screened libraries that did not affect HPV infection and was chosen as an irrelevant negative control compound for the secondary screens. The neutralizing antibody (H16.7E, provided by Neil Christensen at the Pennsylvania State University, Hersey) was used as a positive control for HPV infection inhibition.
