# Supplementary material for: Establishment of Human Papillomavirus Infection Requires Cell Cycle Progression
Source: PLoS Pathog. 2009 Feb 27;5(2):e1000318. doi: 10.1371/journal.ppat.1000318 (PMC2642596; doi:10.1371/journal.ppat.1000318)
Supplement: Figure S2 — Cell cycle progression through M phase is critical for HPV infection. 293T cells were synchronized with aphidicolin (sets 1, 2, 4, and 5) and etoposide (set 3) 4 hrs before wild type HPV16 inoculation. Cell cycle progression was released in set 1 and kept blocked in sets 2 and 3 using aphidicolin and etoposide, respectively. In set 4, cell cycle progressed through S phase but arrested at G2 phase by switching the compounds in medium from aphidicolin to etoposide. In set 5, cell cycle was released for 20 hrs to progress through M phase and arrest at G2 phase by etoposide addition. Wild type HPV16 gene expression was measured from total RNA extracts as indicated in Figure S1 and in Materials and Methods. (0.06 MB PDF) [file ppat.1000318.s002.pdf]

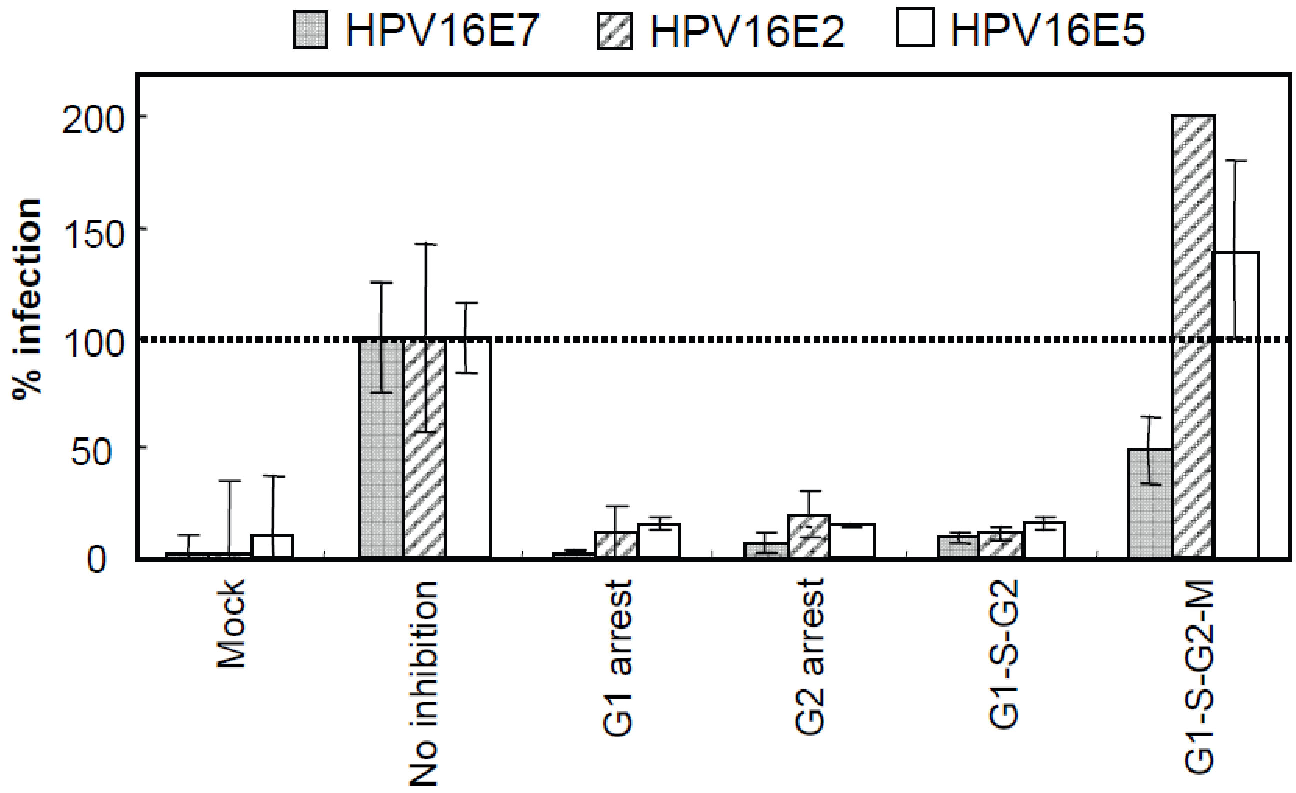


**Supplementary Figure 2.** Cell cycle progression through M phase is critical for HPV infection. 293T cells were synchronized with aphidicolin (sets 1, 2, 4, and 5) and etoposide (set 3) 4 hrs before wild type HPV16 inoculation. Cell cycle progression was released in set 1 and kept blocked in set 2 and 3 using aphidicolin and etoposide, respectively. In set 4, cell cycle progressed through S phase but arrested at G2 phase by switching the compounds in medium from aphidicolin to etoposide. In set 5, cell cycle was released for 20 hrs to progress through M phase and arrest at G2 phase by etoposide addition. Wild type HPV16 gene expression was measured from total RNA extracts as indicated in Supplementary Figure 1 and Experimental Procedures.
