# Supplementary material for: Establishment of Human Papillomavirus Infection Requires Cell Cycle Progression
Source: PLoS Pathog. 2009 Feb 27;5(2):e1000318. doi: 10.1371/journal.ppat.1000318 (PMC2642596; doi:10.1371/journal.ppat.1000318)
Supplement: Figure S3 — Quantitative PCR primers of HPV16 early genes. (A) Oligonucleotide primers were designed using the Primer3 primer design program. (B) The HPV genomic position of each primer is indicated by an arrow. (0.01 MB PDF) [file ppat.1000318.s003.pdf]

A

Primer name	Sequence	Direction	Position
16E7-U	5’-AAATGACAGCTCAGAGGAGGAG	Sense	645-666
16E7-L	5’-GAGTCACACTTGCAACAAAAGG	Antisense	728-749
16E2-U	5’-ACTATCCAGCGACCAAGATCAG	Sense	3464-3485
16E2-L	5’-TGTTAAATGCAGTGAGGATTGG	Antisense	3551-3572
16E5-U	5’-TTTGTGTGCTTTTGTGTGTCTG	Sense	3905-3926
16E5-L	5’-AGAGGCTGCTGTTATCCACAAT	Antisense	3993-4014

B

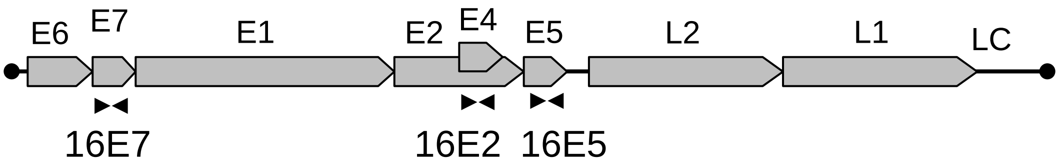

Supplementary Figure 3. Quantitative PCR primers of HPV16 early genes. (A) Oligonucleotide primers were designed using the Primer3 primer design program. (B) The HPV genomic position of each primer is indicated by an arrow.
